# Supplementary material for: COVID-19-Related Burnout and Intention of Fully Vaccinated Individuals to Get a Booster Dose: The Mediating Role of Resilience
Source: Vaccines (Basel). 2022 Dec 27;11(1):62. doi: 10.3390/vaccines11010062 (PMC9860670; doi:10.3390/vaccines11010062)
Supplement: Supplementary file 1 [file vaccines-11-00062-s001.zip › Supplementary Table S1.pdf]

Supplementary Table S1. Corrected item-total correlations for the 13 items of the COVID-19 burnout scale.

**Factors**	**Items**	**Corrected item-total correlations**
**Emotional exhaustion**	1. I feel emotionally tired because of the COVID-19 pandemic	0.682
	2. I feel sad because of the COVID-19 pandemic	0.636
	3. I feel angry because of the COVID-19 pandemic	0.721
	4. I feel frustrated because of the COVID-19 pandemic	0.729
	5. I feel upset because of the COVID-19 pandemic	0.739
**Physical exhaustion**	6. I feel physically tired because of the COVID-19 pandemic	0.726
	7. I have lost my energy because of the COVID-19 pandemic	0.686
	8. I have difficulties in sleep because of the COVID-19 pandemic	0.530
	9. I feel tired in my daily life because of the COVID-19 pandemic	0.703
**Exhaustion due to measures against the COVID-19**	10. I feel tired applying personal protection measures, e.g. wearing a face mask	0.607
	11. I feel tired applying measures in case of COVID-19 symptoms, e.g. rapid tests and PCR tests	0.599
	12. I feel tired applying measures in case of COVID-19 infection, e.g. isolation	0.615
	13. I feel tired of getting vaccinated against coronavirus	0.531
