# Supplementary figures and images for: COVID-19-Related Burnout and Intention of Fully Vaccinated Individuals to Get a Booster Dose: The Mediating Role of Resilience
Source: Vaccines (Basel). 2022 Dec 27;11(1):62. doi: 10.3390/vaccines11010062 (PMC9860670; doi:10.3390/vaccines11010062)

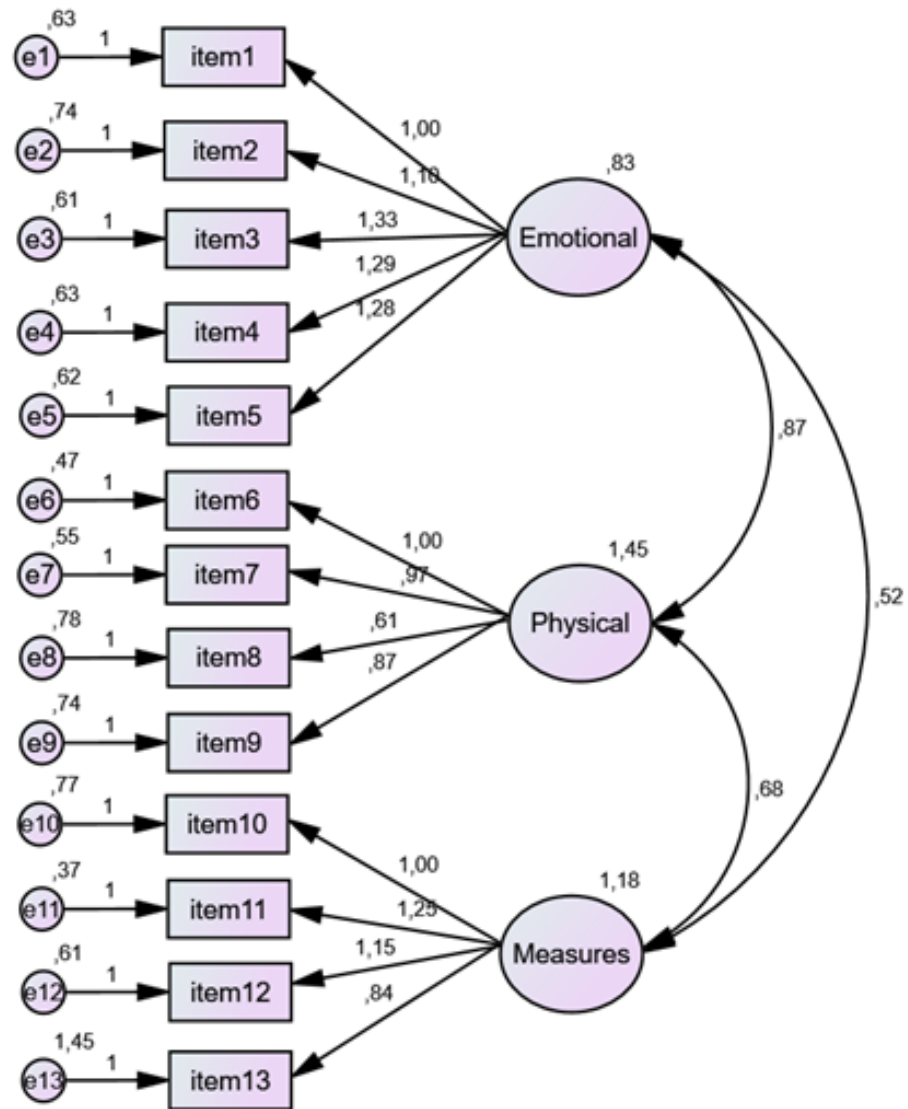

Supplement: Supplementary file 1 [file vaccines-11-00062-s001.zip › Supplementary Figure S1.pdf]
